# Focusing on butterfly eyespot focus: uncoupling of white spots from eyespot bodies in nymphalid butterflies

**DOI:** 10.1186/s40064-016-2969-8

**Published:** 2016-08-08

**Authors:** Masaki Iwata, Joji M. Otaki

**Affiliations:** The BCPH Unit of Molecular Physiology, Department of Chemistry, Biology and Marine Science, Faculty of Science, University of the Ryukyus, Nishihara, Okinawa 903-0213 Japan

**Keywords:** Butterfly, Color pattern, *Calisto tasajera*, Eyespot, Focus, Morphometry, Nymphalidae, Organizing center, White spot

## Abstract

**Background:**

Developmental studies on butterfly wing color patterns often focus on eyespots. A typical eyespot (such as that of *Bicyclus anynana*) has a few concentric rings of dark and light colors and a white spot (called a focus) at the center. The prospective eyespot center during the early pupal stage is known to act as an organizing center. It has often been assumed, according to gradient models for positional information, that a white spot in adult wings corresponds to an organizing center and that the size of the white spot indicates how active that organizing center was. However, there is no supporting evidence for these assumptions. To evaluate the feasibility of these assumptions in nymphalid butterflies, we studied the unique color patterns of *Calisto tasajera* (Nymphalidae, Satyrinae), which have not been analyzed before in the literature.

**Results:**

In the anterior forewing, one white spot was located at the center of an eyespot, but another white spot associated with either no or only a small eyespot was present in the adjacent compartment. The anterior hindwing contained two adjacent white spots not associated with eyespots, one of which showed a sparse pattern. The posterior hindwing contained two adjacent pear-shaped eyespots, and the white spots were located at the proximal side or even outside the eyespot bodies. The successive white spots within a single compartment along the midline in the posterior hindwing showed a possible trajectory of a positional determination process for the white spots. Several cases of focus-less eyespots in other nymphalid butterflies were also presented.

**Conclusions:**

These results argue for the uncoupling of white spots from eyespot bodies, suggesting that an eyespot organizing center does not necessarily differentiate into a white spot and that a prospective white spot does not necessarily signify organizing activity for an eyespot. Incorporation of these results in future models for butterfly wing color pattern formation is encouraged.

## Background

Butterflies and moths are a large group of insects called Lepidoptera. Lepidopteran insects are characterized by wings covered with scales and bristles. These scales are variously colored, and a single scale serves as an image unit (or “pixel”). These scales form diverse mosaic color patterns on wings. One group of butterflies that shows highly diverse color patterns is the family Nymphalidae, from which a common overall color pattern was derived as the nymphalid groundplan (Nijhout 1978, 1991, 2001; Otaki 2009, 2012a; Taira et al. 2015). The nymphalid groundplan is composed of three major symmetry systems (the border, central, and basal symmetry systems) and two peripheral systems (wing root and marginal systems), and all five systems are thought to be produced based on the same mechanism (Otaki 2012a; Taira et al. 2015). A unit of a symmetry system in a single wing compartment is composed of a single core element and a pair of paracore elements located at the distal and proximal sides of the core element (Otaki 2012a).

Among the symmetry systems, the border symmetry system is probably the most conspicuous in many nymphalid butterflies. It is composed of a border ocellus (an eyespot) as a core element and a pair of parafocal elements (distal and proximal parafocal elements) as paracore elements (Nijhout 1991, 2001; Dhungel and Otaki 2009; Otaki 2009, 2012a). Moving from the center to the peripheral area, a typical eyespot is composed of a white focal spot at the center (often called a “focus”), an inner black disk, a light-colored ring, and an outer black ring. A typical eyespot can be found in the African satyrine butterfly, *Bicyclus anynana*, one of the most popular species in butterfly biology (Beldade and Brakefield 2002; Carroll et al. 2004). Physical damage at the prospective eyespot focus in *Junonia coenia* (Nijhout 1980a, 1991), *B. anynana* (French and Brakefield 1992), *Ypthima argus* (Otaki et al. 2005a), *Junonia orithya* (Otaki et al. 2005a), and *Junonia almana* (Otaki 2011a), together with transplantation experiments (Nijhout 1980a, 1991; French and Brakefield 1995; Brakefield et al. 1996; Beldade et al. 2008), demonstrated that the center of the prospective eyespot behaves as an organizing center for the eyespot during the pupal stage. However, actual eyespots are highly diverse, and various deformations from the typical eyespot pattern occur (Nijhout 1990, 1991; Otaki 2011b). For example, the white focal spot is often missing, and the various rings are often distorted differently in a single eyespot.

Since the last decade of the twentieth century, many candidate genes that could specify eyespots have been identified based on their expression patterns (Carroll et al. 1994; Brakefield et al. 1996; Keys et al. 1999; Brunetti et al. 2001; Reed and Serfas 2004; Monteiro et al. 2006; Saenko et al. 2011; Tong et al. 2012). These genes are expressed during the late larval to the early pupal stages in the wing tissues, which is when the color pattern determination takes place (Nijhout 1980a). Among them, the most notable gene is probably *Distal*-*less* (*Dll*). It has been shown that *Dll* expression recapitulates the locations of organizing centers that were predicted by a reaction–diffusion model (Carroll et al. 1994; Nijhout 1990, 1991, 1994, 1996), which has often been interpreted as meaning that *Dll* expression defines an organizing center and that *Dll* is a master gene for eyespot determination. In addition to the eyespot focal determination, it has also been suggested that *Dll* determines eyespot size (Brakefield et al. 1996; Beldade et al. 2002).

However, functional tests for *Dll* were not performed until recently. One study using transgenic *B. anynana* butterflies showed that *Dll* plays a role in eyespot size regulation as well as in black spot induction (Monteiro et al. 2013). One study using the blue pansy butterfly, *J. orithya*, together with a novel surgical technique, showed a weak correlation of the individual *Dll* expression level with the individual eyespot size (Adhikari and Otaki 2016). However, sexually dimorphic eyespot size in this species (i.e., female eyespots are larger than male ones) cannot be explained by the *Dll* expression levels; female forewings have lower *Dll* levels than male ones (Adhikari and Otaki 2016). Subsequently, using *J. orithya* with a baculovirus gene transfer method (Dhungel et al. 2013), it has been shown that *Dll* can induce fragmentary patterns of an eyespot but not an entire eyespot (Dhungel et al. 2016). More elegantly, *Dll* deletion using genome editing has produced a deformation of eyespot, an increase of eyespot number and size, and an emergence of dark patches in *Vanessa cardui* and *J. coenia*, suggesting a role of *Dll* in eyespot repression (Zhang and Reed 2016). Taken together, although *Dll* is unlikely to be sufficient for the entire eyespot pattern formation, it plays an important role in eyespot development.

Morphological studies also advanced. Butterfly wings exhibit coordinated scale size distributions in addition to coordinated scale color distributions (Kusaba and Otaki 2009; Dhungel and Otaki 2013; Iwata and Otaki 2016). The largest scales in an eyespot are often at the central area in *J. orithya* (Kusaba and Otaki 2009) and *J. almana* (Iwata and Otaki 2016). This finding, together with the observation that scale size is proportional to the size of scale-building cells (Henke 1946; Sondhi 1963), led us to propose the ploidy hypothesis that morphogen signals for color patterns are identical to ploidy signals (Iwata and Otaki 2016).

Additionally, the pupal surface has cuticle focal spots that correspond to adult eyespots in various butterfly species (Nijhout 1980a; Otaki et al. 2005a). Two *Junonia* species that have large eyespots in adult wings, *J. orithya* and *J. almana*, indeed have large and distinct pupal cuticle focal spots, whereas a *Junonia* species that has small eyespots in adult wings, *J. hedonia*, has small ones (Taira and Otaki 2016). Interestingly, the size of the cuticle spot is correlated with the size of the corresponding eyespots in *J. orithya* and *Y. argus* (Otaki et al. 2005a). Similar correlations were also obtained among serial eyespots on a single wing in *J. orithya* (Taira and Otaki 2016). The three-dimensional structures of pupal cuticle focal spots as well as adult wings were revealed recently (Taira and Otaki 2016).

Moreover, physiologically induced changes of color patterns, which are typically considered positional and morphological changes of elements, have been investigated in detail (Nijhout 1984; Otaki 1998, 2007, 2008a, b; Otaki and Yamamoto 2004a, b; Serfas and Carroll 2005; Otaki et al. 2005b, 2010; Mahdi et al. 2010, 2011; Hiyama et al. 2012). Meanwhile, an invention of a real-time in vivo observation system made it possible to record how wing tissues develop inside the pupal case (Iwata et al. 2014). Developing epithelial cells are elongated vertically as well as horizontally (Ohno and Otaki 2015a), confirming a century-old histological study (Mayer 1896). Long-range slow calcium waves have been discovered in pupal wing tissues, which may function as signals to coordinate development throughout a wing (Ohno and Otaki 2015b).

This information should collectively evaluate the feasibility of mechanistic models for color pattern determination. Historically, morphogen gradient models have been proposed and used to explain various experimental results (Nijhout 1978, 1980a, 1981, 1990, 1991; French and Brakefield 1992, 1995; Brakefield and French 1995; Monteiro et al. 2001; Serfas and Carroll 2005; Otaki 2008a). Nijhout (1990) examined the diverse eyespot patterns of nymphalid butterflies and identified 36 pattern categories, which were used to construct a gradient-based model. These models are based on the simple diffusion of a putative morphogen that forms a gradient, together with differentiation thresholds inherently programmed into immature scale cells. Abrupt changes of the cellular interpretation of a smooth gradient were attained mathematically by a sigmoidal curve, resulting in two thresholds and three colors (Nijhout 1991).

However, Otaki (2011b, c) pointed out several difficulties of the gradient models to explain actual butterfly wing color patterns. For example, an “archetypical” butterfly eyespot is likely composed of a series of repetitions of an inductive signal for black (or dark) area (Otaki 2011c). In other words, a non-black (i.e., light-colored) area between the black areas is equivalent to background (Otaki 2011c). This binary rule (stating that a series of repetitions of dark areas with light-area intervals is the basic expression of an eyespot) alone makes threshold-based diffusion models unrealistic because the black rings or disks are equivalent to each other in actual butterflies. Moreover, not just two but three or more repetitive black rings are observed in many butterflies (Otaki 2011b). Indeed, one of the “black rings” of an eyespot is a pair of discontinued elements called parafocal elements (Otaki 2009, 2011c, 2012a, b). Moreover, color pattern analysis of neighboring or serial eyespots with different structures on the same wing surface pointed out that thresholds for gradient interpretation, if exist according to the gradient models, do not vary among neighboring compartments and that these eyespots should be produced by different levels of a morphogen to reflect their morphological differences (Otaki 2011b). But it is theoretically difficult to satisfy these two points simultaneously in gradient models (Otaki 2011b). In fact, the dynamic responses of eyespots to physical damage requires flexible models that can accommodate signals from damage sites and from neighboring organizing centers (Otaki 2011a).

As an alternative model, the induction model has been proposed (Otaki 2011b, c, 2012b). The induction model is based on many case analyses of normal and experimentally induced color patterns (Otaki 2011b, c, 2012b), incorporating the principle of “short-range activation and long-range inhibition” that have been found in many biological patterns (Gierer and Meinhardt 1972; Meinhardt 1982; Meinhardt and Gierer 1974, 2000).

In either model, the status of the white focal area has not been explained well in the literature. Nijhout (1978, 1980a) proposed that a “focus” at the center of an eyespot releases a morphogen at the late larval and early pupal stages, based on which a gradient model was formulated. Since then, one tends to assume that the white focal spot directly corresponds to an organizing center for the entire eyespot. In many instances, this assumption seems to be valid; a white spot is located at the physical centers of eyespots in many nymphalid butterflies. However, this is not always the case. Nijhout (1980a) indeed pointed out that the white scales at the eyespot center do not precisely correspond to the “focus”. Likewise, there is a discrepancy between the location of the largest scales and the location of the white spots in a particular eyespot of *J. almana* (Iwata and Otaki 2016). Similar cases have been pointed out in *Calisto herophile* and other butterflies (Iwata and Otaki 2016). Moreover, the white coloration is structural rather than pigment-based (Nijhout 1980b, 1991; Iwata and Otaki 2016). In a gradient model, the area of the highest morphogen concentration above a certain threshold is supposed to become the white spot. But molecular pathways for structural color production are probably qualitatively different from those for pigment-based color production. Thus, one could think that these two production lines may be distinctly specified. In any case, the relationships between white spots and their corresponding eyespot bodies (defined as all the eyespot portions except white spots) should be clarified to understand how butterfly eyespots are constructed during development.

In this paper, we ask if white spots behave independently of eyespot bodies. We hypothesized that if uncoupling of white spots is mechanistically possible, some species of nymphalid butterflies show uncoupling color patterns naturally. More concretely, we hypothesized that it may be possible to observe white spots that are not located at the center of an eyespot in nymphalid butterfly wings and that such uncoupling behavior may be shown by morphometric analysis. Here, we focus on *Calisto* butterflies to test this hypothesis.

Lepidopterists in Asian (and probably in many other) countries are not familiar with the genus *Calisto* because they are endemic to the West Indian regions (mainly in Hispaniola, which is occupied by Haiti and Dominican Republic). Indeed, *Calisto*-type pear-shaped eyespot patterns were not incorporated in the pattern analysis of Nijhout (1990). However, we had an opportunity to examine specimens of *Calisto* butterflies. The genus *Calisto* is an exclusive group of satyrine butterflies in the West Indies that constitutes more than 40 species (Smith et al. 1994; Miller and Miller 2001; Askew and Stafford 2008). Among them, we here focused on eyespots of *Calisto tasajera* González, Schwartz & Wetherbee 1991 (González et al. 1991; Hedges and Johnson 1994) because it has unique pear-shaped eyespots that have two or more white “focal” spots. Molecular phylogenetic analysis and historical biogeography of *Calisto* have been reported (Sourakov and Zakharov 2011; Matos-Maraví et al. 2014). We also examined eyespots of other nymphalid butterflies to support our findings with *C. tasajera*. The present study argues for an uncoupling of white spots from the rest of the eyespots (i.e., eyespot bodies).

## Methods

### Butterflies

**Fig. 1:**
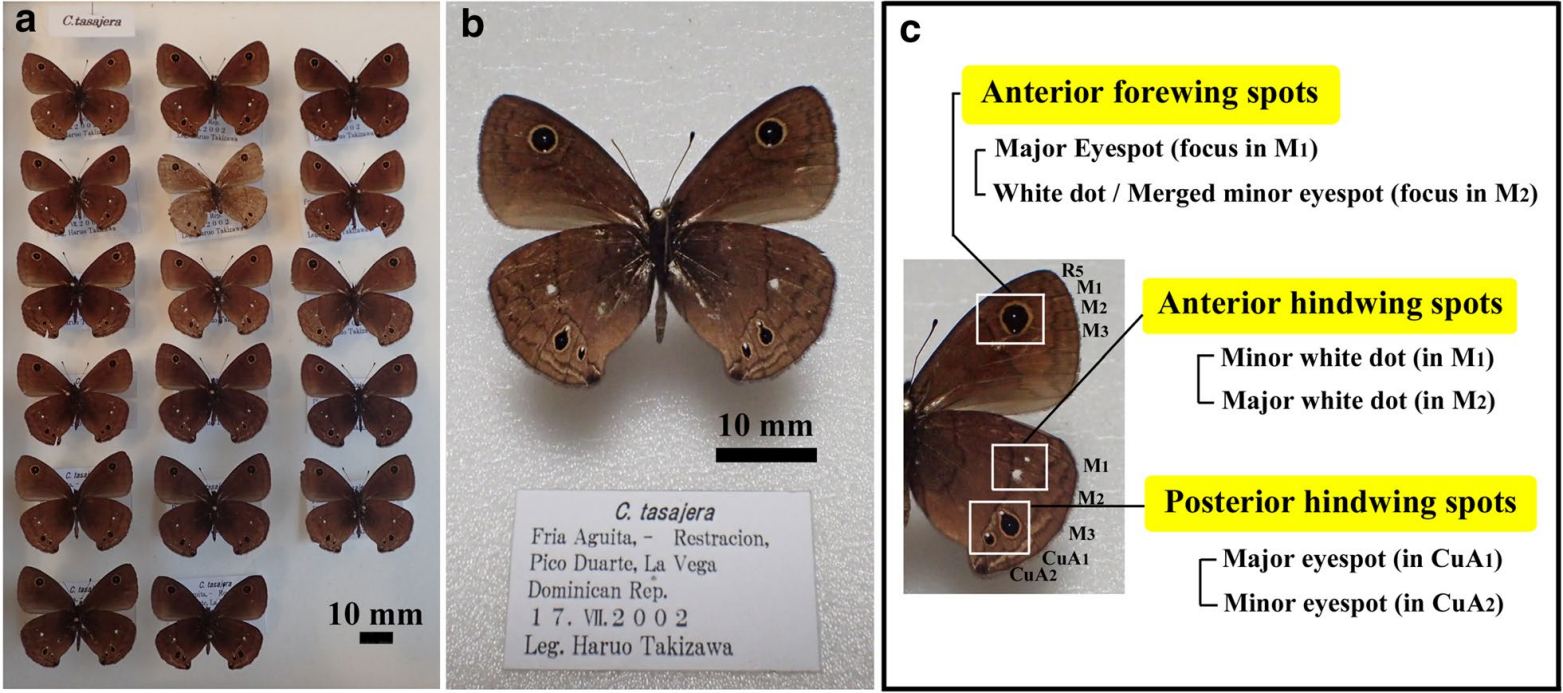
Specimens of *Calisto tasajera*. **a** 17 specimens that were analyzed in this study; **b** a specimen (one of 17) showing the ventral side; **c** three regions of analysis on the ventral wings

We primarily analyzed 17 specimens of *C. tasajera* owned by Nariaki Yamada (The Butterfly Science Society of Japan), Tokyo, Japan (Fig. 1a). These butterflies were collected in the Dominican Republic on July 17, 2002, by Haruo Takizawa (Fig. 1b). Sex of these individuals was not identified; this species is not sexually dimorphic. We focused on three wing regions of the ventral side that contain eyespots and/or white spots: the anterior forewing, the anterior hindwing, and the posterior hindwing (Fig. 1c). The ventral side of the left wings was examined in all cases except for one individual which had left wing damage; in this case, the ventral side of the right wings was examined. Venation patterns of *Calisto* butterflies are not unique. This means that venation patterns do not confer *Calisto* butterflies unique color patterns. Specimens of *Calisto* butterflies (other than *C. herophile*) are owned by N. Yamada. Other butterfly specimens are owned by the author (J. M. O.).

### Images and morphometry

Images of specimens were taken by an Olympus digital camera STYLUS TG-4 Tough (Tokyo, Japan) using its microscope mode. Areas of white spots and black disks of eyespots in *C. tasajera* were measured using ImageJ v. 1.48 image analysis software (Schneider et al. 2012). Because we were not allowed to measure absolute values, which potentially damages the specimens, relative values within a single image were used for comparison in this study.

### Basic assumptions

We assumed that a basic molecular mechanism for color pattern formation is shared in all compartments of a single wing surface or at least in two adjacent wing compartments. This is the very basic assumption that is required for this type of color pattern analysis.

In analyzing color patterns, we assumed that morphogenic signals are released from an organizing center and that the signals move equally well in all directions. These assumptions led to the following interpretations. (1) If an eyespot is close to an exact circle, its signals were released from its physical center. (2) If an eyespot is clearly distorted from an exact circle as observed in an oval or pear-shaped eyespot, its signals were released from two or multiple sites, unless there is a nearby element that blocks the propagation of the morphogenic signals. That is, a morphogenic gradient is made as a merge of two or multiple signals.

In discussing a diffusion-based gradient model, it has often been assumed implicitly that a white spot at the center of an eyespot corresponds to an organizing center for the entire eyespot (see “Background”). We do not believe that this assumption is always correct; however, because this assumption is associated with the gradient models, it was used as a starting point of color pattern analysis in the present study. Furthermore, the simplest form of a conventional diffusion-based gradient model predicts that the eyespot focus had the highest concentration of a morphogen in the larval and pupal wings. Accordingly, the area of the highest morphogen concentration corresponds to a white spot. If the threshold level is fixed and not changeable, the higher the level of morphogen that is released, the wider the area of the white spot that is specified by the morphogen.

In making models for color pattern formation, it is often assumed that adult wing color patterns are directly determined by their pre-patterns in pupal wings. For simplicity, this assumption is also followed in the present study. If one considers that color patterns are finalized through a four-step process (signaling, reception, interpretation, and expression) (Otaki 2008a), a pre-pattern may not be realized solely by the signaling step without the subsequent steps of reception, interpretation, and expression. A direct determination of adult color patterns by pre-patterns thus means that these subsequent steps are all normally executed without positional bias. In reality, however, it has been known that the proximal and distal wing surfaces have different sensitivities to morphogenic signals (Nijhout 1978, 1980a, 1985; Brakefield and French 1995; French and Brakefield 1995).

### Statistics

Numerical values were recorded in Microsoft Excel and analyzed with R statistical software, version 3.2.1 (The R Foundation for Statistical Computing, Vienna, Austria). For each dataset, normality was checked with a Shapiro–Wilk test, based on which nonparametric tests were performed. Mean and standard deviation (SD) values were calculated, and Mann–Whitney *U* tests (pairwise comparison using Wilcoxon rank sum tests) were performed to compare two samples. When multiple pairwise comparisons were made, *p* values were adjusted by Holm correction. The Spearman rank correlation coefficient *ρ* was obtained to examine the possible correlation between two variables.

## Results

### Anterior forewing spots in *C. tasajera*

**Fig. 2:**
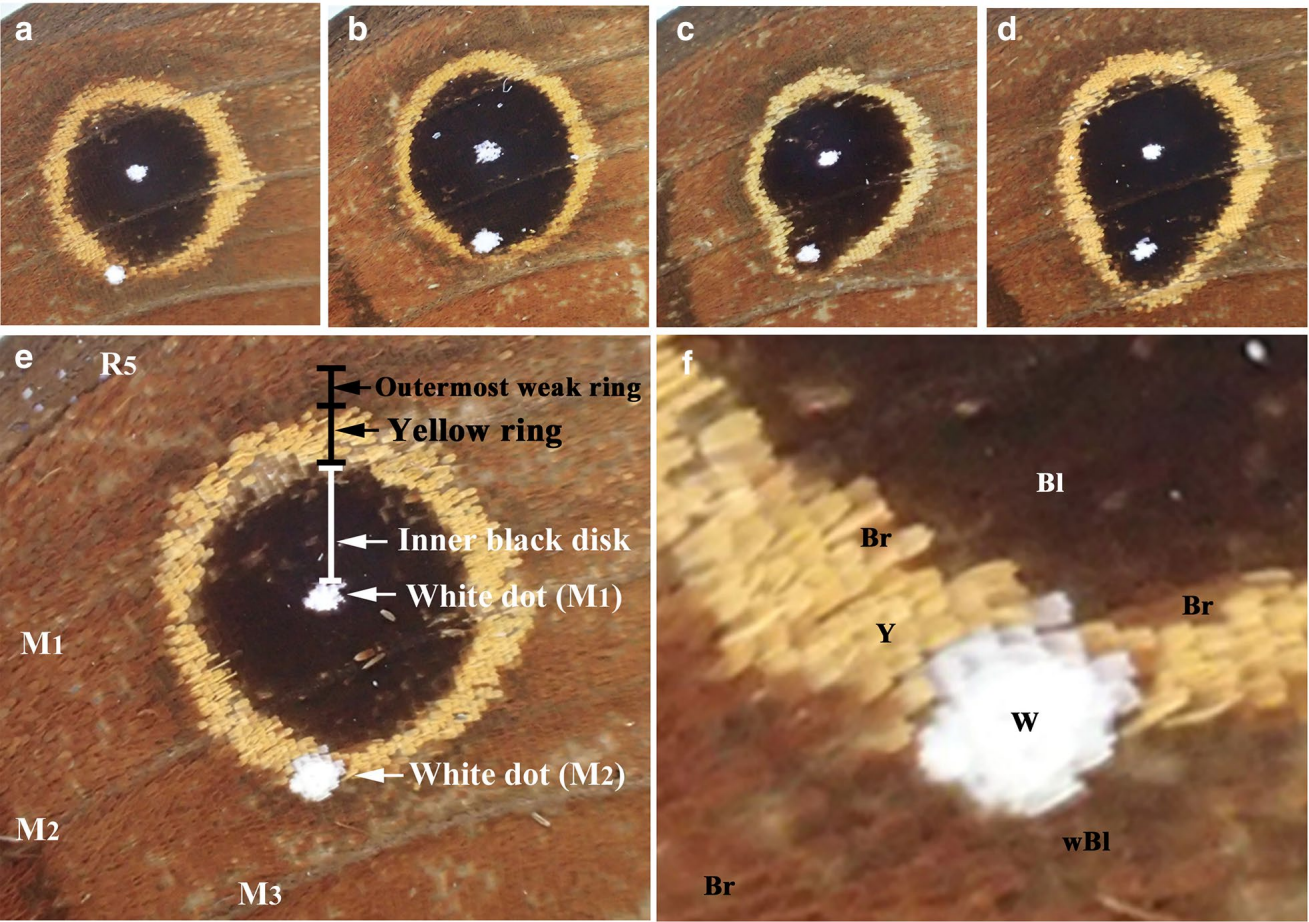
Anterior forewing spots. **a–d** Variations of spots. **e** High magnification image of the anterior forewing spots with annotations. Four wing veins, R_5_, M_1_, M_2_, and M_3_, are marked. **f** Higher magnification image of **e**. *Scale colors* are indicated as follows: W (*white*), Bl (*black*), Y (*yellow*), Br (*brown*), and wBl (*weak black*)

We first analyzed the color patterns of the anterior forewing spots in *C. tasajera*. A relatively large eyespot was present in the anterior forewing (Fig. 2a–f). The white spot (“focus”) of this eyespot was located in the M_1_ compartment, but this eyespot was not confined to the M_1_ compartment; it also occupied two adjacent compartments, R_5_ and M_2_ (Fig. 2). This invading eyespot suggests that immature scale cells in these three compartments were equally receptive to morphogenic signals from the M_1_ organizing center during the late larval and early pupal stages.

In the M_2_ compartment, a white spot was present, the size of which was similar to that of the M_1_ compartment. This white spot was located just on the yellow ring or on the edge of the inner black disk, without a significant distortion of the M_1_ eyespot, in 6 individuals out of 17 (Fig. 2a, e, f). In these cases, the M_1_ eyespot was almost an exact circle. According to a conventional understanding, the central positioning of the white spot within this eyespot suggests that the M_1_ white spot corresponds to the organizing center from which morphogenic signals for the eyespot body were released during development in this particular eyespot. On the other hand, 11 individuals showed a distortion of the M_1_ eyespot; in these cases, the M_2_ white spot was located completely inside the eyespot (Fig. 2b–d). This likely occurs from a fusion of the smaller M_2_ eyespot and the larger M_1_ eyespot. Thus, the M_1_ white spot appears to have corresponded to a highly active organizing center and the M_2_ white spot to either an inactive or less active organizing center.

A close examination of an M_1_ eyespot revealed that there was an outermost weak black ring located outside a yellow ring (Fig. 2e, f). This weak black ring existed all around the yellow ring including the immediate vicinity of the M_2_ white spot in all 6 cases when there was no distortion of the M_1_ eyespot (Fig. 2e, f), suggesting that the M_2_ white spot in these cases was completely inactive as an organizing center for the eyespot body despite a clear expression of the white scales.

### Anterior hindwing spots in *C. tasajera*

**Fig. 3:**
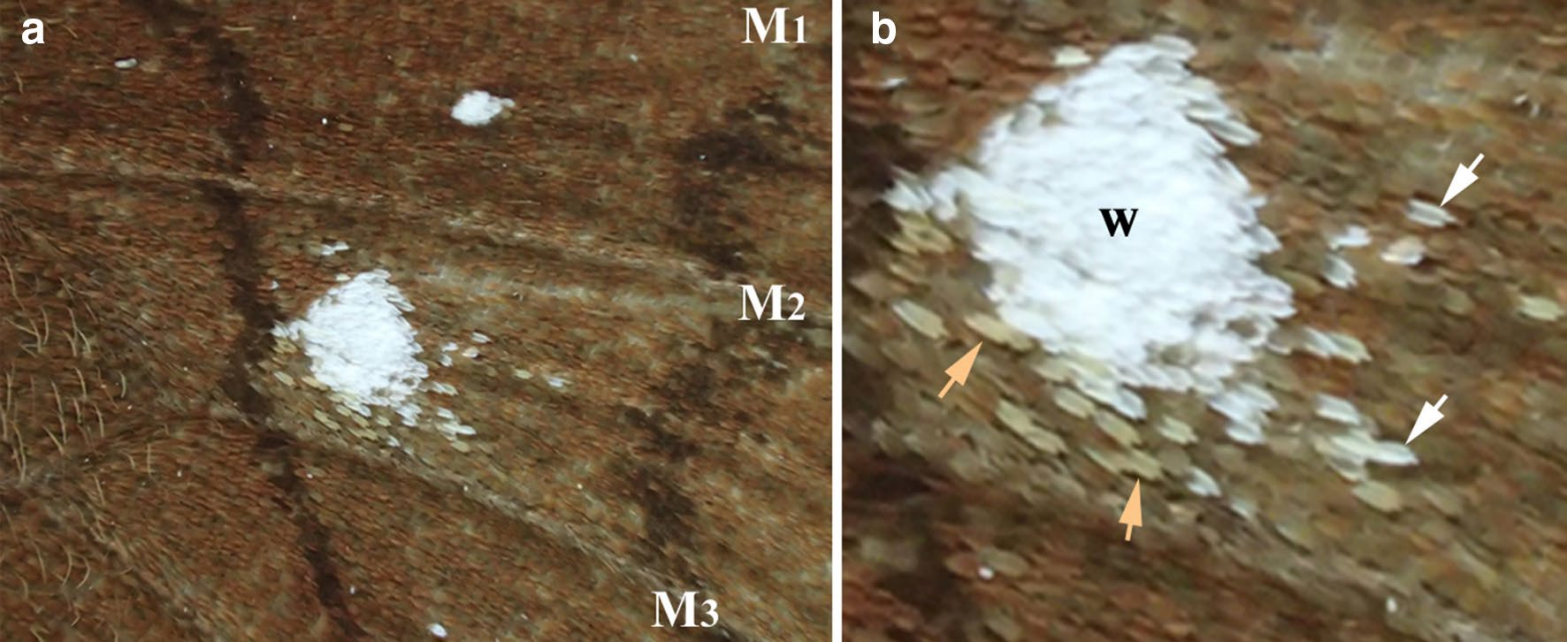
Anterior hindwing spots. **a** White spots in the M_1_ and M_2_ compartments. Three wing veins, M_1_, M_2_, and M_3_, are marked. **b** High magnification of the white spot in the M_2_ compartment. The sparse pattern is clearly observed. Some sparse scales are *white* (*white arrows*), and other sparse scales are *brown* (*light brown arrows*)

The anterior hindwing in *C. tasajera* had two solitary white spots, one in the M_1_ compartment and the other in the M_2_ compartment (Fig. 3). In all 17 individuals, the M_2_ white spot was much larger than the M_1_ white spot (Fig. 3a). Moreover, the M_2_ white spot showed a “sparse pattern” sensu Nijhout (1991). Some of these sparse scales were not white but light brown (Fig. 3b). These scales probably contained relatively small amount of brown pigment and developed white structural color simultaneously, suggesting that a decision-making process for differentiation is not all or nothing. Neither the M_1_ nor the M_2_ white spot accompanied any eyespot structure, suggesting that the organizing cells that differentiated into these white spots did not have any eyespot-inducing activity.

### Posterior hindwing spots in *C. tasajera*

**Fig. 4:**
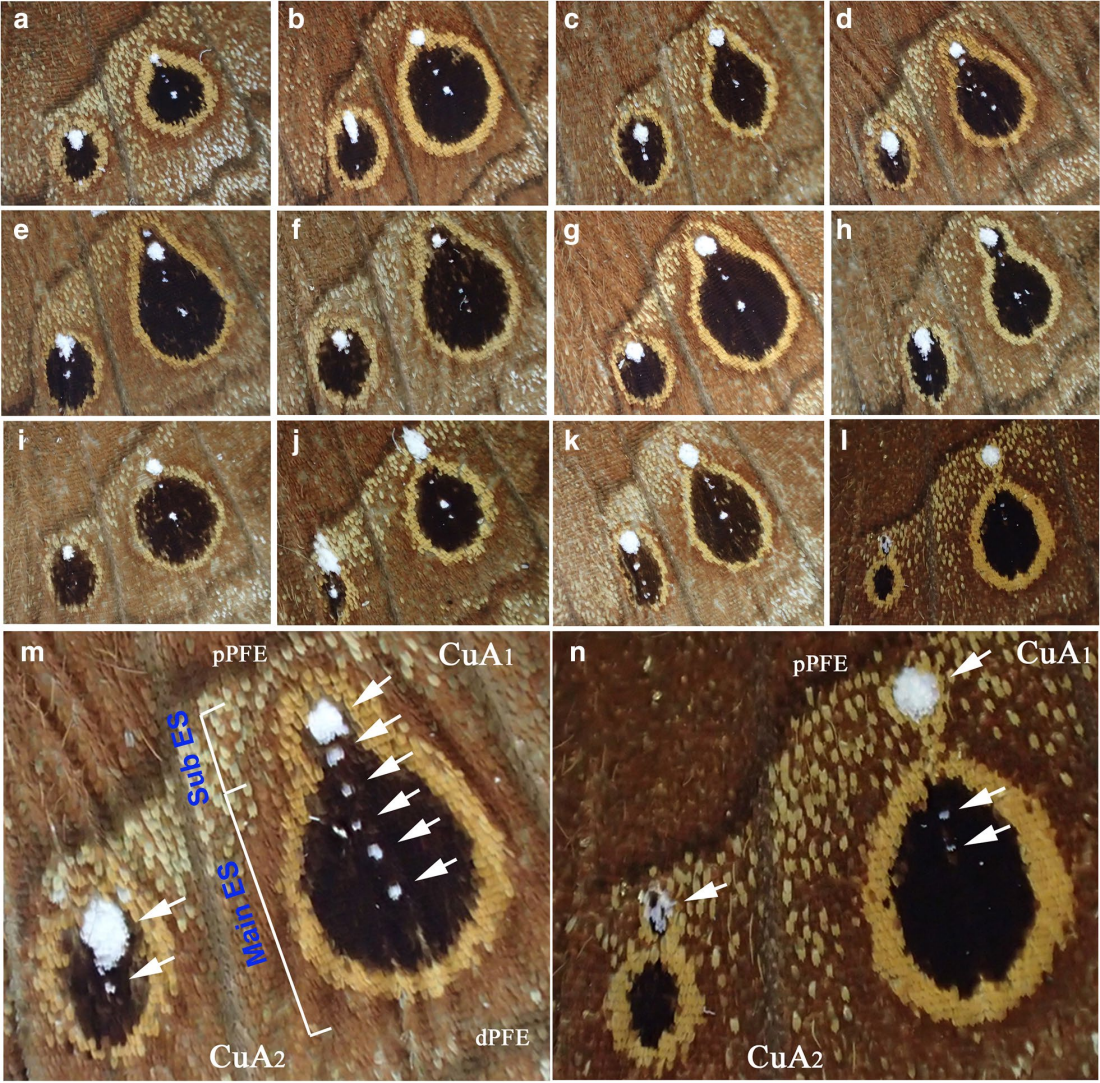
Posterior hindwing spots. **a**–**l** Variations of spots. **m** An example of a high magnification image with annotations. *Arrows* indicate *white dots* along the midline. The *white dots* are located within the merged eyespots. The whole structure may be called a pear-shaped eyespot. The sub eyespot (ES) and the main ES are distinctly named. Wing veins CuA_1_ and CuA_2_ are marked, and parafocal elements, pPFE (proximal PFE) and dPFE (distal PFE) are also indicated (also in **n**). **n** Another example of a high magnification image. *Arrows* indicate white dots along the midline. The *large white dots* are located outside the eyespots in both compartments

The posterior hindwings of *C. tasajera* had two eyespots, one in the CuA_1_ compartment and the other in the CuA_2_ compartment (Fig. 4). Most of the CuA_1_ eyespots (and the CuA_2_ eyespots to a similar degree) were pear-shaped (Fig. 4a–h). These pear-shaped eyespots suggest that morphogenic signals were released from two or more sites within a single compartment. This pear-shaped eyespot morphology can be considered as a merger of two (or more) eyespots: a main eyespot and a sub eyespot (Fig. 4m). In all 17 individuals, the most distinct white spot in these compartments was located at the proximal edge of the eyespot, often with a few small white spots along the midline. The organizing cells for the most proximal white spot did not seem to have been highly active to induce the eyespot body, but the organizing cells for black scales (or the small white dot) at the physical center of the eyespot were probably highly active to induce the eyespot body.

The merger of the main and sub eyespots in the CuA_1_ and CuA_2_ compartments is comparable to that in the anterior forewing region. In the anterior forewing region, two organizing centers (specified by the white spots) were located in different compartments. In the case of the posterior hindwing region, two (or more) organizing centers were located in the same compartment along the midline. Interestingly, in 4 individuals out of 17, the proximal white spot was located outside the eyespot, forming an independent spot (Fig. 4i–l, n).

### Quantitative comparisons of white areas

**Fig. 5:**
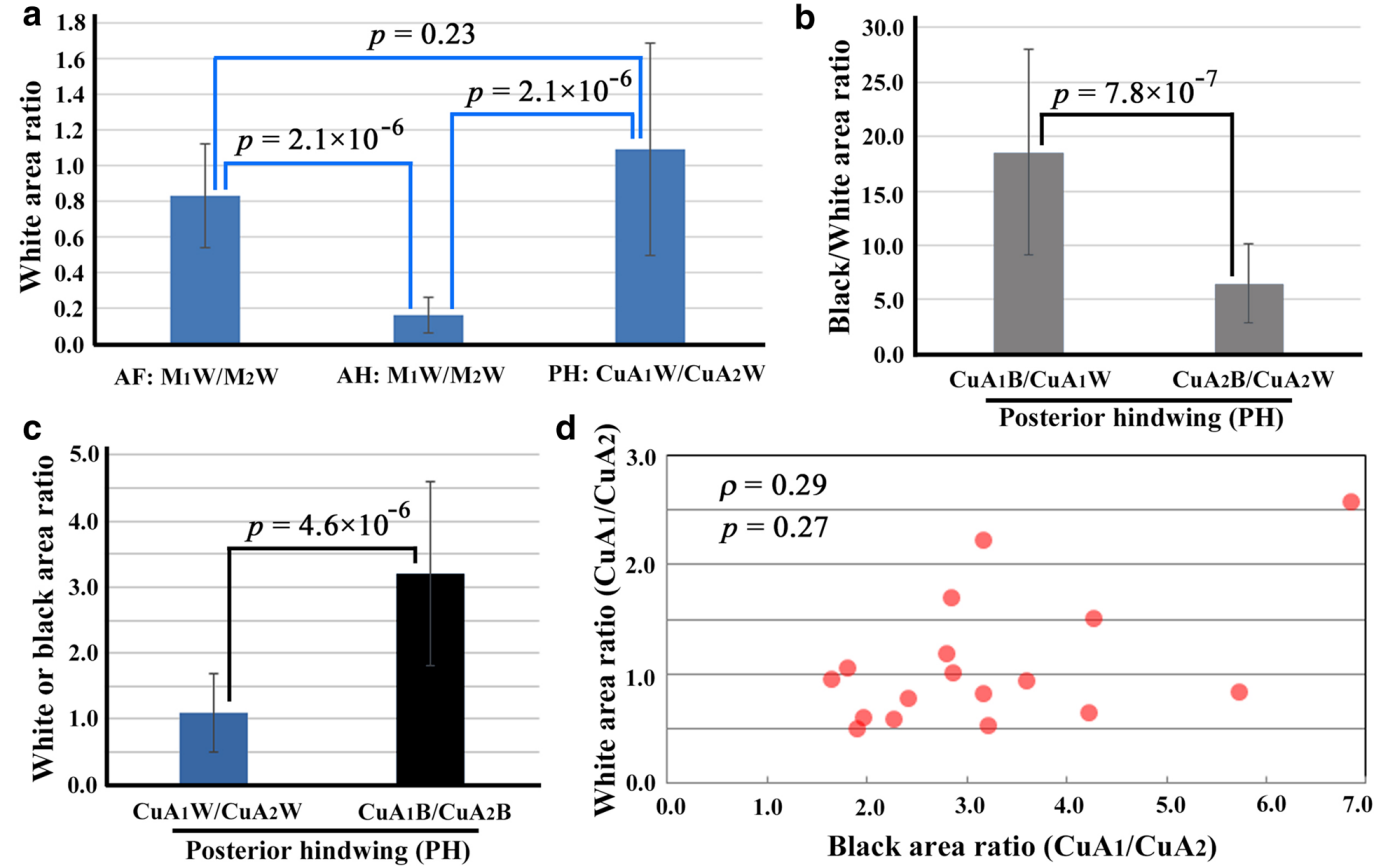
Quantitative comparisons of area ratios in the anterior forewings (AF), anterior hindwings (AH), and posterior hindwings (PH). Compartmental names with W (*white*) or B (*black*) at the end are indicated. **a** *White area* ratios among three wing regions. **b** *Black*/*white* ratios in the posterior hindwing region. **c** *White area* ratio versus *black area* ratio in the posterior hindwing region. **d** Scatter plot of *black area* ratio versus *white area* ratio

The white area ratios were calculated for the anterior forewing region (M_1_/M_2_), the anterior hindwing region (M_1_/M_2_), and the posterior hindwing region (CuA_1_/CuA_2_) of *C. tasajera* (Fig. 5a). Although the anterior forewing and hindwing regions are homologous, the ratios were significantly different between them (*p* = 2.1 × 10^−6^); in the anterior forewing region, the two white dots were similar in size, showing a ratio of 0.83 ± 0.29 (mean ± SD; also hereafter), but in the anterior hindwing region, the ratio was 0.16 ± 0.10. The ratio of the posterior hindwing region was also close to one, 1.09 ± 0.60 and was significantly different from that of the anterior hindwing region (*p* = 2.1 × 10^−6^). The ratios between the anterior forewing region and the posterior hindwing regions were not significantly different (*p* = 0.23).

In the posterior hindwing region, the ratio of black area to white area (black/white) in each compartment was calculated to quantitatively understand eyespot constituents. The ratio of the CuA_1_ compartment, 18.54 ± 9.49, was significantly larger than that of the CuA_2_ compartment, 6.46 ± 3.68 (*p* = 7.8 × 10^−7^) (Fig. 5b). Then, from a different perspective, the ratio of the white areas between the two compartments and the ratio of the black areas between the two compartments were compared. The white area ratio (1.09 ± 0.60) and the black area ratio (3.21 ± 1.4) were significantly different in these two adjacent compartments (*p* = 4.6 × 10^−6^) (Fig. 5c). In 17 individuals, the black area ratio and the white area ratio were not correlated significantly in the Spearman correlation analysis (*ρ* = 0.29; *p* = 0.27) (Fig. 5d).

### White spot diversity in Nymphalidae

Thus far, we have focused on white spot patterns only in *C. tasajera*. To investigate whether similar white spot patterns are present in other species, we examined specimens of other nymphalid butterflies—including other *Calisto* species.

**Fig. 6:**
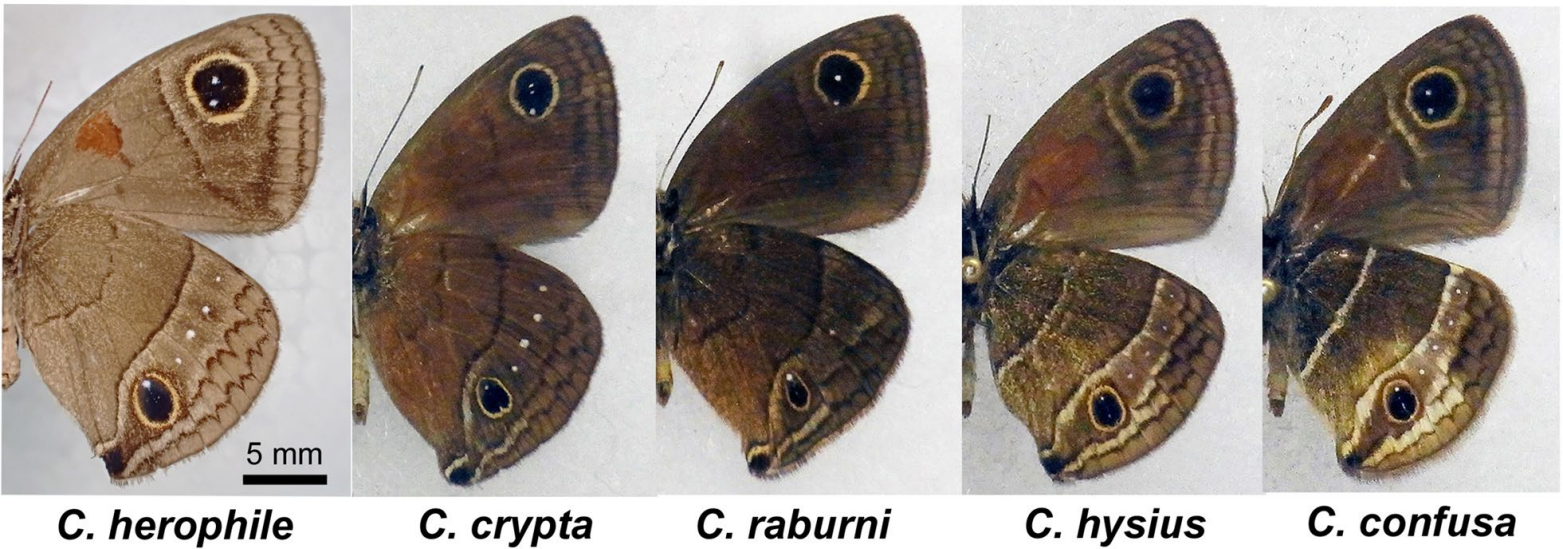
Additional five *Calisto* species. Ventral sides are shown. Images are adjusted so that individuals appear at similar sizes. *Scale bar* 5 mm. This *scale bar* is applicable only to *C. herophile*

We were able to examine five other *Calisto* species (one specimen per species), all of which showed that the eyespots on the posterior hindwing had a proximal white spot with or without multiple small white spots along the midline. In these five species, we were not able to confirm the morphological features that were found in *C. tasajera* in the anterior forewings (i.e., a full circular eyespot in a compartment and a solitary white spot in the adjacent compartment) and anterior hindwings (i.e., two adjacent white spots, one of which shows sparse pattern) (Fig. 6).

**Fig. 7:**
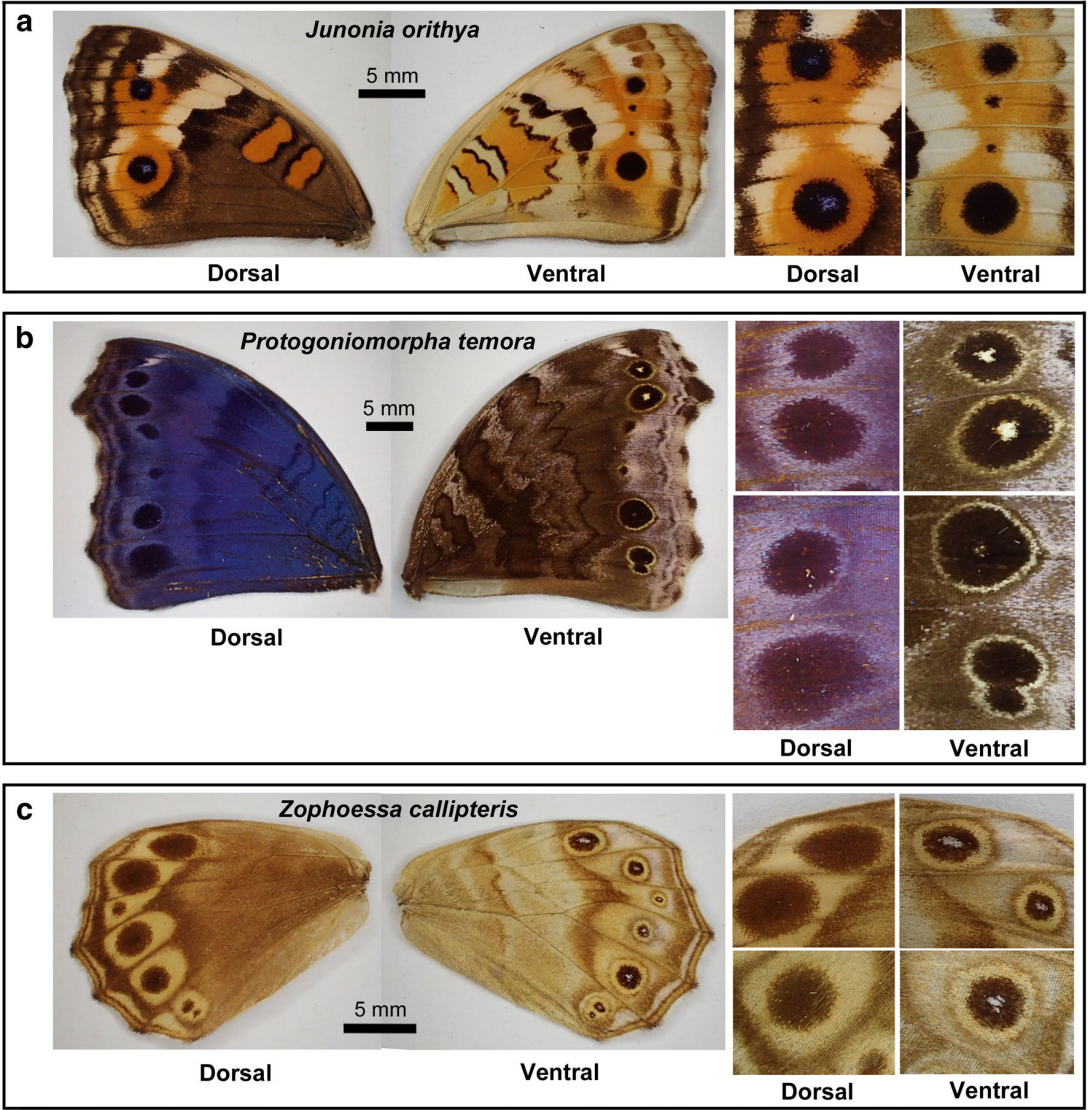
Examples of “focus-less” eyespots. The dorsal and ventral sides are shown. Enlarged eyespot images are shown at the *right side*. *Scale bars* 5 mm. **a** *J. orithya*. **b** *Protogoniomorpha temora*. **c** *Zophoessa callipteris*

A white spot on the proximal side (or even outside) of the main eyespot body, as observed in the posterior hindwings of all the *Calisto* species examined here, is probably not found frequently, but many examples of “focus-less” eyespots were found in other nymphalid butterflies. In the forewings of *J. orithya*, eyespots both on the dorsal and ventral sides were similar in size and structure, but interestingly, the dorsal eyespots had bluish-white spots at the center, whereas the ventral eyespots did not (Fig. 7a). The opposite was true in *Protogoniomorpha temora*: the dorsal eyespots had no white spot, whereas most of the ventral eyespots (not all) had white spots (Fig. 7b).

**Fig. 8:**
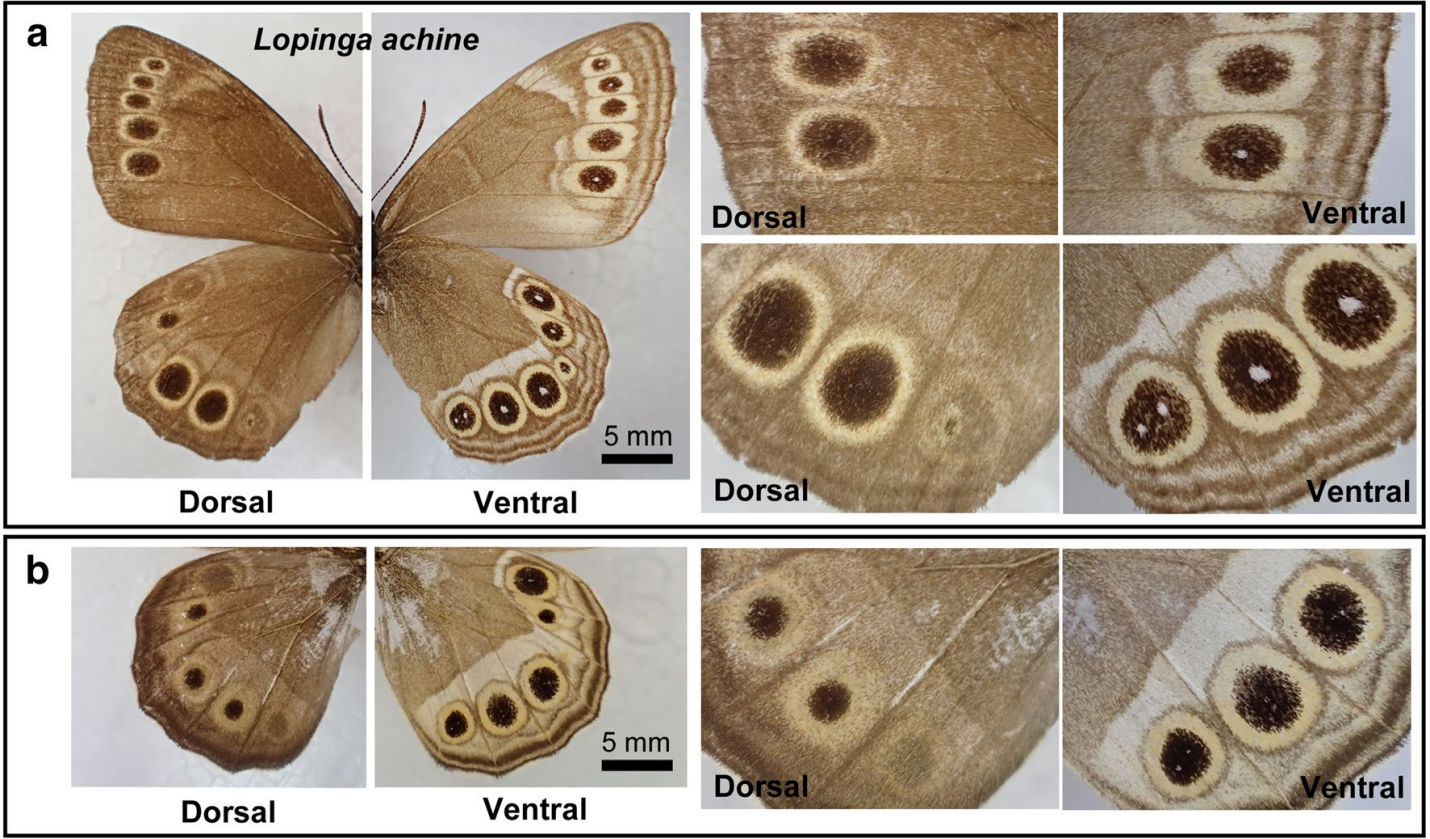
Eyespots of *Lopinga achine*. *Scale bars* 5 mm. **a** Normal wings. Most eyespots (but not all) on the ventral side have a white spot, but those on the dorsal side do not. **b** A mutant wing. The ventral eyespots have no or very small white spots

Many other examples of focus-less eyespots on the dorsal side but not in the ventral side were found in satyrine butterflies, including *Zophoessa callipteris* (Fig. 7c), *Kirinia fentoni*, *Erebia ligea*, *Neope goschkevitschii*, *Neope niphonica*, and *Lopinga achine* (Fig. 8a). Among these, *L. achine* is a noteworthy case: some individuals of this species had focus-less eyespots on both sides of the wings (Fig. 8b). The presence or absence of the white spot did not seem to affect the eyespot size in this species.

## Discussion

### Uncoupling of white spots from eyespot bodies

In this paper, we analyzed the eyespots and white spots of *C. tasajera*. We focused on three regions of ventral wings: the anterior forewing, the anterior hindwing, and the posterior hindwing. In the two adjacent compartments of the anterior forewing, there were a full circular eyespot and a solitary white spot. Also noteworthy is the invasion of the M_1_ eyespot to the adjacent R_5_ and M_2_ compartments, despite the fact that the M_2_ compartment harbors a white spot (with no or small eyespot body) that is as large as that in the M_1_ eyespot, suggesting that all three compartments had the same sensitivity to morphogenic signals. In the anterior hindwing, we observed different shapes and sizes of the white spots in the two adjacent compartments, neither of which were associated with eyespots. In the posterior hindwing, we observed unique pear-shaped eyespots that contained two or more white spots.

Assuming that color patterns are determined by organizing cells, each cluster of organizing cells for white spots and eyespot bodies appear to have behaved differently during development. We speculate that in the anterior forewing, one cluster of organizing cells was highly active and the other was weakly active or completely inactive for eyespot body determination. This means that completely inactive cells (regarding the inducing activity for an eyespot body) can still induce or differentiate into a white spot. If the area values of white spots represent morphogen levels for eyespot bodies, as a conventional gradient model predicts, both the M_1_ and M_2_ compartments in the anterior forewing should have comparable levels of morphogens (indeed, the M_1_/M_2_ ratio of white spots was 0.83, meaning that the M_2_ eyespot is slightly larger than the M_1_ eyespot). In reality, however, the M_2_ white spot is associated with either no or only a small eyespot body (that is, the M_1_/M_2_ ratio of eyespot bodies was infinitely large, meaning that the M_1_ eyespot body is much larger than the M_2_ eyespot body). Therefore, the area values of white spots do not indicate the activity levels of organizing centers for eyespot bodies in the anterior forewing of *C. tasajera*, contrary to the expectation from a conventional gradient model. This conclusion is also relevant for the anterior and posterior hindwing regions in this species (see below).

In the anterior hindwing, there are two different solitary white spots. Interestingly, these two white spots are morphologically different: one is relatively small with clear boundary, and the other is relatively large with diffused boundary, showing the eyespot-independent morphological diversity of white spots. A difference in white patterns in adjacent compartments within the major eyespot of *J. almana* (Iwata and Otaki 2016) is probably a similar phenomenon. Similar white spot patterns were also found in some *Cithaerias* species such as *C.pireta* and in some *Pierella* species such as *P. astyoche* (Otaki 2011b).

In the posterior hindwing, we observed unique pear-shaped eyespots. For the main eyespots of these pear-shaped eyespots, the actual organizing center may be located at the physical center of the main eyespot and may be marked with a small white spot or not marked at all. The large proximal white spots exhibited little or no activity for inducing eyespot bodies. Quantitatively, the area values of white spots were not correlated with the black area values. These results, together with the results of the other two regions, argue that the white-inducing activity is independent of the eyespot-inducing activity—at least in *C. tasajera*. This conclusion was supported by the eyespot and white spot patterns of other *Calisto* butterflies and other nymphalid butterflies.

### Morphological diversity of white spots

Nijhout (1990) examined color patterns of 2208 species (330 genera) of nymphalid butterflies, in which [also in Chapter 7 of Nijhout (1991)] several types of eyespot focal morphology were discussed: arc-shaped foci in *Morpho hecuba*, double foci in *Euptychia*, fragmented foci in *Lethe*, and sparse patterns. Importantly, most of the white spot diverse patterns are successfully reproducible mathematically by a reaction–diffusion model (Nijhout 1990, 1991). Although the white spots of *Calisto* (i.e., the pear-shaped *Calisto*-type eyespots) were not specifically discussed, it was concluded that the relationship between the shape of the white area (or “focus”) and the surrounding ocellus (i.e., eyespot body) is highly variable (Nijhout 1990), which is consistent with the present study. Moreover, Nijhout (1990) introduced “two point sources” along the midline as a part of a “toolbox” to produce diverse eyespot patterns, which is reminiscent of the pear-shaped *Calisto*-type eyespots. It is to be noted that the distortions of the pear-shaped eyespots of *Calisto*, which have two or more foci along the midline, are very different from a common distortion of single-focus eyespots, which was explained by the two-gradient model (Nijhout 1978, 1981).

### Mathematical models

In Chapter 7 of the seminal book (Nijhout 1991) and also in the previous paper (Nijhout 1990), two mathematical models are presented for color pattern formation, one that determines the location of the organizing centers (source formation model) and a second that determines actual eyespots (eyespot formation model). The latter model is based on a morphogen gradient model as discussed in the “Background” section of this paper, while the former is a model for determining the position of the organizing centers (Nijhout 1990, 1991). The former model is given by reaction–diffusion equations based on the principle of “short-range activation and long-range inhibition” (Gierer and Meinhardt 1972; Meinhardt and Gierer 1974, 2000; Meinhardt 1982). In this model, activator concentration becomes high along the midline. This high midline region then retracts toward the wing margin, but a few high activation points are left behind. The multiple white spots along the midline found in the posterior hindwing of *C. tasajera* are therefore compatible with this model. The activator dots will then become white spots in *C. tasajera*. However, stable emergence of the white spots with and without eyespot bodies in particular compartments—as observed in the anterior forewing region in *C. tasajera*—is enigmatic.

Sekimura et al. (2015) recently reported successful simulation of the emergence of an eyespot organizing center in particular compartments but not in other compartments by changing boundary conditions. However, the emerging organizing centers in particular compartments may release morphogenic signals either for an eyespot body or for a white spot or both. In other words, considering the results of the present paper, what is specified in the source formation model (Nijhout 1990, 1991) is the location of immature cells that could differentiate either (1) into a white spot organizing center, or (2) into an eyespot body organizing center (without white spot), or (3) both. It will be interesting to see whether a model similar to that proposed in Sekimura et al. (2015) can explain these various immature-cell-fate options, as observed in the anterior forewing region of *C. tasajera*. Moreover, sparse patterns such as those observed in the anterior hindwing region of *C. tasajera*, which are common in nymphalid eyespots and white spots, should also be simulated in the future.

Genetic network simulations based on expression pattern studies successfully simulated developmental processes and final placement of eyespot focus (Evans and Marcus 2006; Marcus and Evans 2008). The successful simulation results are dependent on careful adjustments of expression thresholds. Evans and Marcus (2006) state that a subtle threshold adjustment could entirely eliminate eyespot development, resulting in an eyespot-less compartment. Furthermore, the irregular shape of eyespot foci in the *comet* and *Cyclops* mutants in *B. anynana* were successfully simulated (Marcus and Evans 2008).

In the eyespot of *B. anynana*, a white spot is placed at the center, signifying the location of organizing center for the entire eyespot. Thus, these simulation studies need no revision (regarding the *B. anynana* eyespot) based on the present results. However, similar but different simulations may be required to differentiate a white spot from an organizing center and to describe the behavior of white spots in *C. tasajera*.

### *Dll* expression in eyespot organizing centers

A high *Dll* expression level is found at the center of prospective eyespots in *J. coenia* [see Fig. 4, page 238 in Brakefield et al. (1996) or Fig. 6.4, page 167 in Carroll et al. (2004)]. The interpretation of this fact has been to consider *Dll* as an important regulatory gene for eyespot formation (Brakefield et al. 1996; Nijhout 1996). However, one should notice that the adult eyespots of *J. coenia* do not have a discrete white “focus” at the center. White scales are scattered along the proximal side of the eyespot. Thus, *Dll* expression appears to regulate eyespot bodies but not to specify the white area in this instance. This interpretation is likely also applicable to the case of *J. almana*, in which the largest scales are blue/black scales located at the central area of the eyespot, while white scales are located proximally (Iwata and Otaki 2016). A similar discussion can be found in Monteiro (2008); the presence or absence of white spot at the center does not signify the presence or absence of focal activity as an eyespot organizing center.

On the other hand, in the case of the dorsal forewing eyespots of *J. orithya*, which have distinct white (or strictly, bluish) focal areas, the size of the entire eyespot as well as the size of these white foci are only weakly correlated with the *Dll* expression level (Adhikari and Otaki 2016). In this case, *Dll* expression that regulates the size of eyespot bodies probably coincides to a certain extent with the expression of unknown genes that regulate the size of white areas. Indeed, white spots are not likely affected much in *Dll*-deleted wings (Zhang and Reed 2016).

### Structural versus pigment-based coloration

The white coloration of white spots is structural, rather than pigment-based, in *Junonia* butterflies (Nijhout 1980b, 1991; Monteiro et al. 2015; Iwata and Otaki 2016). Because developmental processes of structural color production and pigment synthesis would be very different, an uncoupling of the white spots (i.e., structural color expression) from the eyespot bodies (i.e., pigment production) may be reasonable. Interestingly, probably because of the different synthetic pathways, it seems that pigment synthesis and structural color expression are able to coexist in a given cell to some extent, because brownish white scales are present in the anterior hindwing region in *C. tasajera*. Furthermore, colored foci are not rare in nymphalid butterflies. A good example is the dorsal forewing eyespots of *J. orithya*, which have bluish white foci. The coexistence of structural and pigment-based coloration in focal spots certainly contributes to the diversity of “white spots” in nymphalid butterflies.

## Conclusions

Eyespot body behavior and white spot behavior are different and separable, although the same cells may function to organize both in many instances. The size of white spots in adults does not necessarily reflect the degrees of organizing activity for eyespots. Because white coloration of white scales is structural rather than pigment-based, the differentiation mechanism for white scales may be independent from that for black or other pigmented scales.
